# 2013 WSES guidelines for management of intra-abdominal infections

**DOI:** 10.1186/1749-7922-8-3

**Published:** 2013-01-08

**Authors:** Massimo Sartelli, Pierluigi Viale, Fausto Catena, Luca Ansaloni, Ernest Moore, Mark Malangoni, Frederick A Moore, George Velmahos, Raul Coimbra, Rao Ivatury, Andrew Peitzman, Kaoru Koike, Ari Leppaniemi, Walter Biffl, Clay Cothren Burlew, Zsolt J Balogh, Ken Boffard, Cino Bendinelli, Sanjay Gupta, Yoram Kluger, Ferdinando Agresta, Salomone Di Saverio, Imtiaz Wani, Alex Escalona, Carlos Ordonez, Gustavo P Fraga, Gerson Alves Pereira Junior, Miklosh Bala, Yunfeng Cui, Sanjay Marwah, Boris Sakakushev, Victor Kong, Noel Naidoo, Adamu Ahmed, Ashraf Abbas, Gianluca Guercioni, Nereo Vettoretto, Rafael Díaz-Nieto, Ihor Gerych, Cristian Tranà, Mario Paulo Faro, Kuo-Ching Yuan, Kenneth Yuh Yen Kok, Alain Chichom Mefire, Jae Gil Lee, Suk-Kyung Hong, Wagih Ghnnam, Boonying Siribumrungwong, Norio Sato, Kiyoshi Murata, Takayuki Irahara, Federico Coccolini, Helmut A Segovia Lohse, Alfredo Verni, Tomohisa Shoko

**Affiliations:** 1 Department of Surgery, Macerata Hospital, Macerata, Italy; 2 Clinic of Infectious Diseases, Department of Internal Medicine Geriatrics and Nephrologic Diseases, St Orsola-Malpighi University Hospital, Bologna, Italy; 3 Emergency Surgery, Maggiore Parma Hospital, Parma, Italy; 4 Department of General Surgery, Ospedali Riuniti, Bergamo, Italy; 5 Department of Surgery, Denver Health Medical Center, Denver, CO, USA; 6 American Board of Surgery, Philadelphia, PA, USA; 7 University of Texas Health Science Center, Houston, TX, USA; 8 Harvard Medical School, Division of Trauma, Emergency Surgery and Surgical Critical Care Massachusetts General Hospital, Boston, MA, USA; 9 Department of Surgery, UC San Diego Health System, San Diego, CA, USA; 10 Department of Surgery, Virginia Commonwealth University Medical Center, Richmond, VA, USA; 11 Division of General Surgery, University of Pittsburgh Medical Center, Pittsburgh, PA, USA; 12 Department of Primary Care & Emergency Medicine, Kyoto University Graduate School of Medicine, Kyoto, Japan; 13 Department of Abdominal Surgery, University Hospital Meilahti, Helsinki, Finland; 14 Department of Surgery, University of Newcastle, Newcastle, NSW, Australia; 15 Department of Surgery, Charlotte Maxeke Johannesburg Hospital University of the Witwatersrand, Johannesburg, South Africa; 16 Department of Surgery, Govt Medical College and Hospital, Chandigarh, India; 17 Department of General Surgery, Rambam Health Care Campus, Haifa, Israel; 18 Department of Surgery, Adria Hospital, Adria, Italy; 19 Department of Surgery, Maggiore Hospital, Bologna, Italy; 20 Department of Digestive Surgery Faculty of Medicine Pontificia Universidad Católica de Chile, Santiago, Chile; 21 Department of Surgery, Sheri-Kashmir Institute of Medical Sciences, Srinagar, India; 22 Department of Surgery, Universidad del Valle, Fundacion Valle del Lili, Cali, Colombia; 23 Division of Trauma Surgery, Hospital de Clinicas - University of Campinas, Campinas, Brazil; 24 Emergency Unit, Department of Surgery, Ribeirão Preto, Brazil; 25 Department of General Surgery, Hadassah Medical Center, Jerusalem, Israel; 26 Department of Surgery, Tianjin Nankai Hospital, Nankai Clinical School of Medicine, Tianjin Medical University, Tianjin, China; 27 Department of Surgery, Pt BDS Post-graduate Institute of Medical Sciences, Rohtak, India; 28 First Clinic of General Surgery, University Hospital /UMBAL/ St George Plovdiv, Plovdiv, Bulgaria; 29 Department of Surgery, Edendale Hospital, Pietermaritzburg, Republic of South Africa; 30 Department of Surgery, Port Shepstone Hospital, Kwazulu Natal, South Africa; 31 Department of Surgery, Ahmadu Bello University Teaching Hospital Zaria, Kaduna, Nigeria; 32 Department of Surgery, Mansoura University Hospital, Mansoura, Egypt; 33 Department of Surgery, Mazzoni Hospital, Ascoli Piceno, Italy; 34 Department of Surgery, Mellini Hospital, Chiari (BS), Italy; 35 Department of General and Digestive Surgery, University Hospital, Malaga, Spain; 36 Department of General Surgery, Lviv Emergency Hospital, Lviv, Ukraine; 37 Department of Surgery, Ancona University, Ancona, Italy; 38 Division of General and Emergency Surgery, Faculdade de Medicina da Fundação do ABC, São Paulo, Santo André, Brazil; 39 Department of Surgery, Chang Gung Memorial Hospital, Taoyuan, Taiwan; 40 Department of Surgery, Ripas Hospital, Bandar Seri Begawan, Brunei; 41 Clinical Sciences, Regional Hospitals Limbe and Buea, Limbe, Cameroon; 42 Department of Surgery, Severance Hospital, Yonsei University College of Medicine, Seoul, Republic of Korea; 43 Division of Trauma and Surgical Critical Care, Department of Surgery, University of Ulsan, Seoul, Republic of Korea; 44 Wagih Ghnnam, Department of Surgery, Khamis Mushayt General Hospital, Khamis Mushayt, Saudi Arabia; 45 Boonying Siribumrungwong, Department of Surgery, Thammasat University Hospital, Pathumthani, Thailand; 46 Department of Acute and Critical Care Medicine, Tokyo Medical and Dental University, Tokyo, Japan; 47 Department of Emergency and Critical Care Medicine, Nippon Medical School, Emergency and Critical Care Center of Nippon Medical School, Tama-Nagayama Hospital, Tokyo, Japan; 48 II Catedra de Clinica Quirúrgica, Hospital de Clínicas, San Lorenzo, Paraguay; 49 Department of Surgery, Cutral Co Clinic, Neuquen, Argentina; 50 The Shock Trauma and Emergency Medical Center, Matsudo City Hospital, Chiba, Japan

## Abstract

Despite advances in diagnosis, surgery, and antimicrobial therapy, mortality rates associated with complicated intra-abdominal infections remain exceedingly high.

The 2013 update of the World Society of Emergency Surgery (WSES) guidelines for the management of intra-abdominal infections contains evidence-based recommendations for management of patients with intra-abdominal infections.

## Introduction

The clinical recommendations discussed in these guidelines are based on research conducted by members of the WSES Expert Panel. These updated guidelines replace those previously published in 2010 [1]. The guidelines outline clinical recommendations based on the Grading of Recommendations Assessment, Development, and Evaluation (GRADE) hierarchy criteria summarized in Table 1[2,3].

**Table 1:** **Grading of recommendations from Guyatt and colleagues [**[1]**,**[2]**]**

**Grade of recommendation**	**Clarity of risk/benefit**	**Quality of supporting evidence**	**Implications**
**1A**			
Strong recommendation, high-quality evidence	Benefits clearly outweigh risk and burdens, or vice versa	RCTs without important limitations or overwhelming evidence from observational studies	Strong recommendation, applies to most patients in most circumstances without reservation
**1B**			
Strong recommendation, moderate-quality evidence	Benefits clearly outweigh risk and burdens, or vice versa	RCTs with important limitations (inconsistent results, methodological flaws, indirect or imprecise) or exceptionally strong evidence from observational studies	Strong recommendation, applies to most patients in most circumstances without reservation
**1C**			
Strong recommendation, low-quality or very low-quality evidence	Benefits clearly outweigh risk and burdens, or vice versa	Observational studies or case series	Strong recommendation based on limited evidence; recommendations may change when higher quality or more extensive evidence becomes available
**2A**			
Weak recommendation, high-quality evidence	Benefits closely balanced with risks and burdens	RCTs without important limitations or overwhelming evidence from observational studies	Weak recommendation, best action may differ depending on circumstances, expertise of clinician, the patient in question, or other social issues
**2B**			
Weak recommendation, moderate-quality evidence	Benefits closely balanced with risks and burdens	RCTs with important limitations (inconsistent results, methodological flaws, indirect or imprecise) or exceptionally strong evidence from observational studies	Weak recommendation, best action may differ depending on circumstances, expertise of clinician, the patient in question, or other social issues
**2C**			
Weak recommendation, Low-quality or very low-quality evidence	Uncertainty in the estimates of benefits, risks, and burdens; benefits, risks, and burdens may be closely balanced	Observational studies or case series	Very weak recommendation; other alternatives may be equally reasonable

### Principles of surgical management

Intra-abdominal infections (IAIs) encompass a variety of pathological conditions, ranging from uncomplicated appendicitis to fecal peritonitis [4].

As a general principle, every verified source of infection should be controlled as soon as possible. The level of urgency of treatment is determined by the affected organ(s), the relative speed at which clinical symptoms progress and worsen, and the underlying physiological stability of the patient.

The procedure used to treat the infection depends on the anatomical site of infection, the degree of peritoneal inflammation, the generalized septic response, the patient’s underlying condition, and the available resources of the treatment center. IAIs are subcategorized in 2 groups: uncomplicated and complicated IAIs [5].

In the event of an uncomplicated case of IAI, the infection involves a single organ and does not spread to the peritoneum. Patients with such infections can be treated with either surgical intervention or antibiotics. When the infection is effectively resolved by means of surgery, a 24-hour regimen of perioperative antibiotics is typically sufficient. Patients with uncomplicated intra-abdominal infections, such as acute diverticulitis, acute cholecystitis, and acute appendicitis, may be treated non-operatively by means of antimicrobial therapy.

In the event of complicated IAI, the infectious process proceeds beyond a single organ, causing either localized or diffuse peritonitis. The treatment of patients with complicated intra-abdominal infections involves both surgical and antibiotic therapy [5].

The safety and efficacy of ultrasound- and CT-guided percutaneous drainage of abdominal abscesses has been documented in patients with appendiceal and diverticular abscesses. Percutaneous image-guided drainage may also be used to address cases of advanced acute cholecystitis.

### Sepsis management

Patients with severe sepsis or septic shock of abdominal origin require early hemodynamic support, source control, and antimicrobial therapy (Recommendation 1A).

Abdominal sepsis occurs as result of intra-abdominal or retroperitoneal infection. Early detection of the site of infection and timely therapeutic intervention are crucial steps for improving the treatment outcome of sepsis patients.

Sepsis is a complex, multifactorial, evolutive syndrome that can progress to conditions of varying severity. If improperly treated, it may cause the functional impairment of one or more vital organs or systems, which could lead to multiple organ failure [6]. Previous studies have demonstrated that there is an increased risk of death as patients transition from sepsis to severe sepsis and septic shock [7]. In the context of intra-abdominal infections, severe sepsis represents the diagnostic threshold separating stable and critical clinical conditions.

Thus, early detection of severe sepsis and prompt, aggressive treatment of the underlying organ dysfunction is an essential component of improving patient outcome. If untreated, sepsis dysfunction can lead to global tissue hypoxia, direct tissue damage, and ultimately to multiple organ failure [8-10].

Sepsis in the surgical patient continues to be a common and potentially lethal problem. Early identification of patients and timely implementation of evidence-based therapies continue to represent significant clinical challenges for care providers. The implementation of a sepsis screening program in conjunction with protocol for the delivery of evidence-based care and rapid source control can improve patient outcomes [11].

Early, correctly administered resuscitation can improve the outcome of patients with severe sepsis and septic shock (Recommendation 1A).

Rivers et al. demonstrated that a strategy of early goal-directed therapy (EGDT) decreases the in-hospital mortality of patients admitted to the emergency department in septic shock [9].

In surgical patients early intervention and implementation of evidence-based guidelines for the management of severe sepsis and septic shock improve outcomes in patients with sepsis [12].

Patients with severe sepsis and septic shock may present with inadequate perfusion. Poor tissue perfusion can lead to global tissue hypoxia and, in turn, to elevated levels of serum lactate. Fluid resuscitation should be initiated as early as possible in patients with severe sepsis.

The Surviving Sepsis Campaign guidelines [10] recommend that fluid challenge in patients with suspected hypovolemia begin with >1000 mL of crystalloids or 300–500 mL of colloids administered over a period of 30 minutes. Quicker administration and greater volumes of fluid may be required for patients with sepsis-induced tissue hypoperfusion. Given that the volume of distribution is smaller for colloids than it is for crystalloids, colloid-mediated resuscitation requires less fluid to achieve the same results. A colloid equivalent is an acceptable alternative to crystalloid, though it should be noted that crystalloids are typically less expensive.

When fluid challenge fails to restore adequate arterial pressure and organ perfusion, clinicians should resort to vasopressor agents. Vasopressor drugs maintain adequate blood pressure and preserve perfusion pressure, thereby optimizing blood flow in various organs.

Both norepinephrine and dopamine are the first-line vasopressor agents to correct hypotension in septic shock. Both norepinephrine and dopamine can increase blood pressure in shock states, although norepinephrine seems to be more powerful. Dopamine may be useful in patients with compromised cardiac function and cardiac reserve [13], but norepinephrine is more effective than dopamine in reversing hypotension in patients with septic shock. Dopamine has also potentially detrimental effects on the release of pituitary hormones and especially prolactin, although the clinical relevance of these effects is still unclear and can have unintended effects such as tachyarrhythmias. Dopamine has different effects based on the doses [14].

A dose of less than 5 μg/kg/min results in vasodilation of renal, mesenteric, and coronary districts. At a dose of 5–10 μg/kg/min, beta-1-adrenergic effects increase cardiac contractility and heart rate. At doses about 10 μg/kg/min, alpha-adrenergic effects lead to arterial vasoconstriction and increase blood pressure. Its major side effects are tachycardia and arrhythmogenesis.

The use of renal-dose dopamine in sepsis is a controversial issue. In the past, low-dose dopamine was routinely used because of the possible renal protective effects. Dopamine at a dose of 2–3 μg/kg/min was known to stimulate diuresis by increasing renal blood flow.

A meta-analysis of literature from 1966 to 2000 for studies addressing the use of dopamine in the prevention and/or treatment of renal dysfunction [15] concluded that the use of low-dose dopamine for the treatment or prevention of acute renal failure was not justified on the basis of available evidence.

Norepinephrine is a potent alpha-adrenergic agonist with minimal beta-adrenergic agonist effects. Norepinephrine can successfully increase blood pressure in patients who are septic and remain hypotensive following fluid resuscitation. Norepinephrine is effective to treat hypotension in septic shock patients. In many studies norepinephrine administration at doses 0.01 to 0.3 μg/kg/min has been shown may be effective [16,17].

Martin and coll. [18] published a randomized trial comparing norepinephrine vs dopamine. 32 volume-resuscitated septic patients were given either dopamine or norepinephrine to achieve and maintain normal hemodynamic and oxygen transport parameters for at least 6 h. Dopamine administration was successful in only 31% of patients, whereas norepinephrine administration was successful in 93%. Of the 11 patients who did not respond to dopamine, 10 responded when norepinephrine was added to therapy. Serum lactate levels were decreased as well, suggesting that norepinephrine therapy improved tissue oxygenation.

Recently a prospective trial by Patel and coll. compared dopamine to norepinephrine as the initial vasopressor in fluid resuscitated 252 adult patients with septic shock [19]. If the maximum dose of the initial vasopressor was unable to maintain the hemodynamic goal, then fixed dose vasopressin was added to each regimen. If additional vasopressor support was needed to achieve the hemodynamic goal, then phenylephrine was added. In this study dopamine and norepinephrine were equally effective as initial agents as judged by 28-day mortality rates. However, there were significantly more cardiac arrhythmias with dopamine treatment.

The Surviving Sepsis Campaign guidelines [10] state that there is no sufficient evidence to suggest which agent is better as initial vasopressor in the management of patients with septic shock.

Phenylephrine is a selective alpha-1 adrenergic receptor agonist primarily used in anesthesia to increase blood pressure. Although studies are limited [20], its rapid onset, short duration, and primary vascular effects make it an important agent in the management of sepsis-associated hypotension; however, it should be noted that there are concerns regarding its potential to reduce cardiac output in certain patients.

Epinephrine is a potent α-adrenergic and β-adrenergic agent that increases mean arterial pressure by increasing both cardiac index and peripheral vascular tone. The primary concern regarding the use of epinephrine in septic patients is its potential to decrease regional blood flow, particularly in the splanchnic circulation [21].

Vasopressin infusion of 0.01 to 0.04 U/min in patients with septic shock increases plasma vasopressin levels to those observed in patients with hypotension attributable to other etiologies, such as cardiogenic shock. Increased vasopressin levels are associated with a reduced demand for other vasopressors. Urinary output may increase, and pulmonary vascular resistance may decrease. Infusions >0.04 U/min may lead to adverse, vasoconstriction-mediated events [22]. Low doses of vasopressin (0.03 U/min) may be effective in raising blood pressure in patients refractory to other vasopressors and may convey other therapeutic benefits.

Dobutamine is frequently used to treat septic shock patients as an inotropic agent that increases cardiac output, stroke index, and oxygen delivery (Do_2_). However, the tendency of dobutamine to increase Do_2_ to supranormal values in critically ill patients has raised serious questions regarding its saftey in the treatment of septic shock. The Surviving Sepsis Campaign Guidelines [10] recommend that a dobutamine infusion be administered in the event of myocardial dysfunction as indicated by elevated cardiac filling pressures and low cardiac output

The clinical benefits of corticosteroids in the treatment of severe sepsis and septic shock remain controversial.

A systematic review of corticosteroids in the treatment of severe sepsis and septic shock in adult patients was recently published in which the authors discussed 17 randomized trials (2138 patients) and 3 quasi-randomized trials (n = 246) of acceptable methodological quality and pooled the results in a subsequent meta-analysis [23]. The authors concluded that corticosteroid therapy has been used in varied doses for treating sepsis and related syndromes for more than 50 years, but its ability to reduce mortality rates has never been conclusively proven. Since 1998, studies have consistently used prolonged low-dose corticosteroid therapy, and follow-up analyses of this subgroup have found that such regimens tend to reduce short-term mortality.

According to the findings of the meta-analysis, corticosteroids should be considered at daily doses of 200–300 mg of hydrocortisone (or equivalent), administered as either an intravenous bolus or continuous infusion. Although the evidence supporting this claim was not particularly robust, the authors nevertheless suggested that treatment be administered at full dosage for at least 100 hours in adult patients presenting with vasopressor-dependent septic shock.

### Diagnosis

Early diagnosis and prompt treatment of intra-abdominal infections can minimize complications (Recommendation 1C).

Detection of complicated intra-abdominal infections is primarily a clinical diagnosis. However, critically ill patients may be difficult to evaluate due to distracting injuries, respiratory failure, obtundation, or other comorbidities.

Initially, the pain may be dull and poorly localized (visceral peritoneum) before progressing to steady, severe, and more localized pain (parietal peritoneum).

Signs of hypotension and hypoperfusion such as lactic acidosis, oliguria, and acute alteration of mental status are indicative of a patient’s transition to severe sepsis.

Diffuse abdominal rigidity suggests peritonitis and should be addressed promptly by means of aggressive resuscitation and surgical intervention.

Plain films of the abdomen are often the first imaging analyses obtained for patients presenting with intra-abdominal infections.

Upright films are useful for identifying free air beneath the diaphragm (most often on the right side) as an indication of perforated viscera.

The diagnostic approach to confirming the source of abdominal infection in septic patients depends largely on the hemodynamic stability of the patient [24].

For unstable patients who do not undergo an immediate laparotomy and whose critical condition prevents them from leaving the ICU for further imaging analysis, ultrasound is the best available imaging modality (Recommendation 1B).

**For stable, adult patients who do not undergo an immediate laparotomy, computerized tomography (CT) is the imaging modality of choice for diagnosing intra-abdominal infections. In children and young adults, exposure to CT radiation is of particular concern and must be taken into consideration (Recommendation 1B)**.

When patients are stable, computerized tomography (CT) is the optimal imaging modality for assessing most intra-abdominal conditions [24,25].

When possible, computed tomography (CT) of the abdomen and pelvis is the most effective means of diagnosing intra-abdominal infections.

The value of both CT imaging and ultrasound in the diagnostic work-up of intra-abdominal infections has been comprehensively studied in the context of acute appendicitis.

In 2006, a meta-analysis by Doria et al. demonstrated that CT imaging featured significantly higher sensitivity and resolution than ultrasound in studies of both children and adults with acute appendicitis [26].

However, when examining children and young adults, clinicians must always take into account the risk of radiation exposure associated with CT.

Although CT scans are very useful in a clinical setting, children are more radiosensitive than adults and their exposure to ionizing radiation should be minimized [27].

Recently, a single-blind, noninferiority trial, evaluated the rate of negative (unnecessary) appendectomies following low-dose and standard-dose abdominal CTs in young adults with suspected appendicitis. Low-dose CTs were noninferior to standard-dose CTs with respect to negative appendectomy rates in young adults with suspected appendicitis [18]. However low-dose CTs could not detect perforated viscera as effectively as their standard-dose counterparts.

When CT and abdominal ultrasound are not available diagnostic options, diagnostic peritoneal lavage may be useful for the diagnosis of complicated IAIs [24].

### Acute appendicitis

The appendectomy remains the treatment of choice for acute appendicitis. Antibiotic therapy is a safe means of primary treatment for patients with uncomplicated acute appendicitis, but this conservative approach is less effective in the long-term due to significant recurrence rates. (Recommendation 1A).

Although the standard treatment for acute appendicitis has historically been the appendectomy, the medical community has recently seen a notable increase in the use of antibiotic therapy as a primary means of treatment.

Several meta-analyses have been published overviewing a series of randomized trials comparing antibiotic therapy to appendectomies for acute uncomplicated appendicitis (cases without abscesses or phlegmon) [28-31].

Although non-operative, antibioitic-mediated treatments of uncomplicated appendicitis are associated with significantly fewer complications, more manageable pain control, and shorter patient sick leave, this conservative approach features high rates of recurrence and is therefore inferior to the traditional appendectomy.

Considering that only a small number of RCTs of poor methodological quality are currently available, well-designed RCTs are required to better assess the effects of an antibiotic-based approach in conservative treatments of uncomplicated acute appendicitis.

Given this controversy, the appendectomy remains the treatment of choice for acute appendicitis. Non-operative antibiotic treatment may be used as an alternative treatment for specific patients for whom surgery is contraindicated.

Both open and laparoscopic appendectomies are viable approaches to surgical treatment of acute appendicitis (Recommendation 1A).

Several randomized trials have compared the diagnostic and therapeutic advantages of laparoscopic and conventional open appendectomies in the treatment of acute appendicitis.

While the trials demonstrated a reduction in wound infections for the laparoscopic appendectomy group, they also exhibited a threefold increase in intra-abdominal abscesses.

In 2010, Sauerland et al. updated a previously published meta-analysis comparing the diagnostic and therapeutic results of laparoscopic and conventional open surgery [32]. 56 studies comparing laparoscopic appendectomies (with or without diagnostic laparoscopy) to open appendectomies for adult patients were included in the meta-analysis. Wound infections were less likely following a laparoscopic appendectomy (LA) than they were following an open appendectomy (OA), but the laparoscopic procedure showed an increased prevalence of intra-abdominal abscesses. The duration of surgery was on average 10 minutes longer for LAs that it was for open procedures. Compared to OAs, LAs typically resulted in less post-operative pain; on day 1 after surgery, patients who underwent a laparoscopic procedure reported reduced pain by 8 mm on a 100 mm visual analogue scale compared to patients who had undergone the open procedure. Further, the overall hospital stay was reduced for patients who underwent LAs compared to those who underwent OAs. While the operational costs of LAs were significantly higher, the costs associated with recovery were substantially reduced. 7 studies of children were included in the review, but the results did not differ significantly from those of similar adult-focused studies. Diagnostic laparoscopy reduced the risk of unnecessary appendectomies, though this trend was most common in fertile women as compared to unselected adults [33].

However, in many cases the strong predictive power of CT and ultrasound analysis renders the diagnostic laparoscopy clinically superfluous.

In 2011, Masoomi et al. used the Nationwide Inpatient Sample Database to evaluate the clinical data of adult patients in the United States who had undergone either LAs or OAs for suspected acute appendicitis from 2006 to 2008 [34].

A total of 573,244 adults underwent emergency appendectomies during this 3-year period. Overall, 65.2% of all appendectomies were performed laparoscopically. Use of the laparoscopic approach increased 23.7% from 58.2% in 2006 to 72% in 2008. In the context of acute non-perforated appendicitis, LAs featured lower overall complication rates, lower in-hospital mortality rates, and a shorter mean length of hospitalization compared to the open procedure.

**Routine use of intraoperative irrigation for appendectomies does not prevent intra-abdominal abscess formation, adds extra costs, and may be avoided (Recommendation 2B)**.

Recently a retrospective review of 176 consecutive appendectomies, open (39%) and laparoscopic (61%), at a university affiliated tertiary care facility from July 2007 to November 2008 investigated routine use of intraoperative irrigation for appendectomies. The results did not show decrease in postoperative intra-abdominal abscess with use of intraoperative irrigation. Thirteen patients developed postoperative abscess: 11 with irrigation, two without irrigation. Ten of 13 patients who developed abscess were perforated; nine with irrigation and one without [35].

Patients with periappendiceal abscesses should be treated with percutaneous image-guided drainage. (Recommendation 1B).

**Current evidence demonstrates that an interval appendectomy is not routinely necessary following initial non-operative treatment of complicated appendicitis. However, interval appendicectomies should always be performed for patients with recurrent symptoms (Recommendation 2B)**.

For patients with acute appendicitis presenting with abscesses, the optimal management strategy is somewhat controversial.

Percutaneous drainage to address periappendiceal abscesses results in fewer complications and shorter overall hospitalization [36-38].

In 2010, a meta-analysis was published comparing conservative treatment (i.e., antibiotic therapy +/− percutanteous abscess drainage) to appendectomies in the treatment of complicated appendicitis (cases exhibiting abscesses or phlegmon) [39].

17 studies (16 non-randomized/retrospective and 1 non-randomized/prospective) reported clinical data for 1572 patients: 847 patients received conservative treatment and 725 underwent acute appendectomies. Conservative treatment was associated with significantly fewer complications, wound infections, abdominal/pelvic abscesses, ileal/bowel obstructions, and additional follow-up surgeries. No significant differences were found in the overall length of hospitalization or in the duration of intravenous antibiotic infusion. Overall, several clinical studies demonstrated that there were significantly fewer complications in the conservative treatment group than there were in the appendectomy group.

The authors concluded that conservative treatment of complicated appendicitis was associated with decreased complication rates and fewer repeat surgeries (“re-operations”) compared to traditional appendectomies, while both treatments featured comparable lengths of hospitalization.

Traditional management is initially conservative followed by interval appendectomies performed after resolution of the mass.

Recently, the efficacy of interval appendicectomies has been called into question, and there is disagreement in the medical community regarding whether or not the procedure is appropriate for adults with appendiceal abscesses. The main dispute involves the recurrence and complication rates following interval appendectomies as well as the procedure’s ability to address underlying malignancy. The literature provides little evidence that an interval appendicectomy is routinely necessary; findings instead demonstrate that the procedure is unnecessary in 75%-90% of cases [40-42]. The results of a review by Andersonn and Petzold [41] based primarily on retrospective studies supported the practice of nonsurgical treatment without interval appendectomies in patients with appendiceal abscesses or phlegmon. Appendiceal abscesses or phlegmon were found in 3.8% of patients with appendicitis. Nonsurgical treatment failed in 7.2% of these cases, and abscess drainage was required in 19.7%. Immediate surgery was associated with higher morbidity rates compared to nonsurgical treatment. After successful nonsurgical treatments, malignancy and serious benign diseases were detected in 1.2% and 0.7% of cases, respectively, during follow-up analyses.

Following successful conservative treatment, interval appendicectomies were only performed for patients with recurrent symptoms. In patients over 40 years of age, other pathological causes of right iliac masses could be excluded by means of further investigation (colonoscopy and computerized tomography scans).

Studies investigating interval appendectomies after conservative treatment of appendiceal masses are typically retrospective in nature. The risk of recurrence of symptoms is only 7.2%, which suggests that appendectomies may not be routinely necessary [29]. Due to significant variability between studies and their retrospective natures, additional studies are needed to confirm these findings.

### Diverticulitis

**Patients with uncomplicated acute diverticulitis should be treated with antibiotic therapy to address gram-negative and anaerobic pathogens (Recommendation 2C)**.

The routine use of antibiotics for patients with uncomplicated acute diverticulitis is a point of controversy in the medical community.

In 2011, a systematic review was published overviewing antibiotic use in cases of uncomplicated diverticulitis [43]. Relevant data regarding the use of antibiotics in mild or uncomplicated cases of diverticulitis were sparse and of poor methodological quality. There was no concrete evidence to support the routine use of antibiotics in the treatment of uncomplicated diverticulitis.

Recently a prospective, multicenter, randomized trial involving 10 surgical departments in Sweden and 1 in Iceland investigated the use of antibiotic treatment in cases of acute uncomplicated diverticulitis. Antibiotic treatment for acute uncomplicated diverticulitis neither accelerated recovery nor prevented complications or recurrence [44].

However, even in the absence of evidence supporting the routine use of antibiotics for patients with uncomplicated acute diverticulitis, we recommend adequate antimicrobial coverage for gram-negative and anaerobic microorganisms.

Mild cases of uncomplicated acute diverticulitis should be treated in an outpatient setting. Outpatient treatment of uncomplicated acute diverticulitis depends on the condition and compliance of the patient as well as his or her availability for follow-up analysis. The treatment involves orally administered antibiotics to combat gram-negative and anaerobic bacteria. If symptoms persist or worsen, the patient should be admitted for more aggressive inpatient treatment.

Hospitalized patients with uncomplicated acute diverticulitis should be treated with intravenous fluids and antibiotic infusion.

The clinical value of antibiotics in the treatment of acute uncomplicated left-sided diverticulitis is poorly understood by the medical community and therefore merits further study.

The grade and stage of diverticulitis are determined by clinical severity and Hinchey classification of disease, and used to identify patents likely to fail medical management or require surgery. Hinchey's classification provides a means of consistent classification of severity of disease for clinical description and decision making. Perforation with operative findings of purulent peritonitis corresponds to Hinchey stage III, and feculent peritonitis to Hinchey stage IV. Stage I and Stage II refer to inflammatory phlegmon and paracolic abscesses [45].

**Systemic antibiotic treatment alone is usually the most appropriate treatment for patients with small (< 4 cm in diameter) diverticular abscesses; image-guided (ultrasound- or CT-guided) percutaneous drainage is suggested for patients with large diverticular abscesses (> 4 cm in diameter) (Recommendation 2B)**.

For patients with diverticulitis complicated by peridiverticular abscesses, the size of an abscess is an important factor in determining the proper course of action and in deciding whether or not percutaneous drainage is the optimal approach [46].

Patients with small pericolic abscesses (< 4 cm in diameter) without generalized peritonitis (Hinchey Stage 1) can be treated conservatively with bowel rest and broad-spectrum antibiotics [47].

For patients with peridiverticular abscesses larger than 4 cm in diameter, observational studies indicate that CT-guided percutaneous drainage is the treatment of choice [48-51].

**Recommendations for elective sigmoid colectomy following recovery from acute diverticulitis should be made on a case-by-case basis (Recommendation 1C)**.

The role of prophylactic surgery following conservatively managed diverticulitis remains unclear and controversial.

Although elective resection is often recommended after single episodes of complicated acute diverticulits that were resolved with conservative treatment, such an invasive procedure following a favorable response to noninvasive methods has serious implications and should be made on an individual basis [52-55].

Acute diverticulitis has a low rate of recurrence and rarely progresses to more serious complications, and as such, elective surgery to prevent recurrence and development of further complications should be used sparingly.

To investigate recurrence rates and post-operative complications following conservatively managed diverticulitis, Eglinton et al. retrospectively analyzed clinical data from all patients with diverticulitis admitted to their department from 1997 to 2002 [56]. After an initial episode of uncomplicated diverticulitis, only 5% of patients went on to develop the complicated form of the disease. Complicated diverticulitis recurred in 24% of patients, compared to a recurrence rate of 23.4% in those with uncomplicated diverticulitis. Recurrence typically occurred within 12 months of the initial episode.

Recently, Makela et al. published a review of 977 patients admitted for acute diverticulitis during a 20-year period [57]. The authors found that even with 2 or more previous admissions for acute diverticulitis, sigmoid resection remained unjustifiably excessive.

Elective surgery is recommended for patients with pelvic abscesses treated by means of percutaneous drainage due to the poor long-term outcomes of conservative treatment. However, minor mesocolic abscesses that typically resolve when treated conservatively are not always grounds for surgical intervention (Recommendation 1B).

Given the poor outcomes of pelvic abscesses associated with acute left-sided colonic diverticulitis, percutaneous drainage followed by secondary colectomy is recommended [58].

In the event of a colectomy performed to address diverticular disease, a laparoscopic approach is appropriate for select patients (Recommendation 1B).

Laparoscopic colectomies may have some advantages over open colectomies, including less post-operative pain, fewer cosmetic considerations, and a shorter average length of hospitalization. However, there appears to be no significant difference in early or late complication rates between the laparoscopic and open procedures [59,60].

The cost and outcome of the laparoscopic approach are both comparable to those of the open resection [61].

Laparoscopic surgery is recommended for elderly patients [62] and appears to be safe for select patients with complicated diverticulitis [63].

Emergency surgery is required for patients with acute diverticulitis associated with diffuse peritonitis as well as for patients with acute diverticulitis whose initial non-operative management has failed (Recommendation 1B).

Hartmann’s resection is recommended in the event of severe acute diverticulitis with generalized, purulent, or fecal peritonitis as well as for patients with poor prognostic criteria. In the event of diffuse peritonitis, resection with primary anastomosis and peritoneal lavage is a suitable approach for patients with promising prognostic criteria or for those whose non-operative management of acute diverticulitis has failed.

Hartmann’s procedure has historically been the standard treatment for complicated acute diverticulitis [64].

However, bowel reconstruction following Hartmann’s procedure requires additional surgeries, which many patients cannot undergo due to complicated medical conditions; therefore, many of these patients remain with permanent stoma [65].

The optimal approach for treating left colonic perforation is a one-stage procedure involving primary anastomosis.

In an emergency setting, intraoperative lavage of the colon and primary anastomosis are safe procedures for addressing complicated diverticulitis, though Hartmann’s procedure is still recommended for cases of diffuse or fecal peritonitis, immunocompromised patients, or patients experiencing septic shock and multiorgan failure [66].

Many studies have demonstrated that, for select patients, primary anastamosis can be safely performed in the presence of localized or diffuse peritonitis [67].

Primary anastomosis is not recommended for patients in high-risk categories [67-73].

In 2010, Tabbara et al. reviewed the medical records of 194 patients with complicated acute diverticulitis from 1996 to 2006 who required a colectomy within 48 hours of hospital admission [74].

The independent criteria predictive of eventual resection with primary anastomosis included the following: age less than 55 years, period between hospital admission and surgery lasting longer than 4 hours, and a Hinchey score of I or II.

There were patients featuring many of these indicators of primary anastomosis who instead underwent fecal diversion. The conditions and characteristics of these patients were comparable to those of the primary anastomosis patients, yet the former group experienced poorer clinical outcomes than the latter.

In the event of either intraoperative difficulty or extraperitoneal anastomosis, a diverting loop ileostomy following resection and primary anastomosis ,may suggested for high-risk patients who are hemodynamically stable; in this case, high risk is defined by immunosuppression, fecal peritonitis, and/or ASA grade IV [71].

Masoomi et al. [75] using the National Inpatient Sample database, examined the clinical data of patients who underwent an urgent open colorectal resection (sigmoidectomy or anterior resection) for acute diverticulitis from 2002 to 2007 in the United States. A total of 99,259 patients underwent urgent surgery for acute diverticulitis during these years [Primary anastomosis without diversion: 39.3%; Hartman's procedure (HP): 57.3% and primary anastomosis with proximal diversion (PAD): 3.4%]. The overall complication rate was lower in the PAD group compared with the HP group (PAD: 39.06% vs. HP: 40.84%; p = 0.04). Patients in the HP group had a shorter mean length of stay (12.5 vs.14.4 days, p < 0.001) and lower mean hospital costs (USD 65,037 vs. USD 73,440, p < 0.01) compared with the PAD group. Mortality was higher in the HP group (4.82 vs. 3.99%, p = 0.03).

PAD improved outcomes compared with HP, and should be considered in patients who are deemed candidates for two-stage operations for acute diverticulitis.

Laparoscopic peritoneal lavage with placement of drainage tubes is a safe approach for cases of perforated diverticulitis (Recommendation 2B).

Several case series and prospective studies have demonstrated that laparoscopic peritoneal lavage is a safe alternative to conventional management in the treatment of perforated diverticulitis with diffuse purulent peritonitis [76-79].

Recently a retrospective population study used an Irish national database to identify patients acutely admitted with diverticulitis, was published. Demographics, procedures, comorbidities, and outcomes were obtained for the years 1995 to 2008 [80].

Two thousand four hundred fifty-five patients underwent surgery for diverticulitis, of whom 427 underwent laparoscopic lavage. Patients selected for laparoscopic lavage had lower mortality (4.0% vs 10.4%, p < 0.001), complications (14.1% vs 25.0%, p < 0.001), and length of stay (10 days vs 20 days, p < 0.001) than those requiring laparotomy/resection. Patients older than 65 years were more likely to die (OR 4.1, p < 0.001), as were those with connective tissue disease (OR 7.3, p < 0.05) or chronic kidney disease (OR 8.0, p < 0.001).

### Colonic carcinoma perforation

Patients with perforated colonic carcinoma represent the highest risk cases of colonic perforation [81].

Treatments for perforated colonic carcinoma should both stabilize the emergency condition of the peritonitis and fulfil the technical objectives of oncological intervention (Recommendation 1B).

Treating perforated colorectal cancer is a complicated procedure and the prognosis is rarely straightforward. Colorectal cancer-induced perforation is considered an advanced stage disease due to the potential for peritoneal dissemination of tumor cells throughout the site of perforation [82].

The stage of illness, proximity of the perforation to the tumor, and the number of metastatic lymph nodes are positively correlated with reduced procedural and cancer-free survival rates [83].

Hartmann's procedure has been widely accepted as an effective means of treating carcinoma of the left colon (with adequate R0 resection) in certain emergency scenarios [84].

A diverting ileostomy is recommended when anastomosis is performed for high-risk patients.

### Colonic perforation following colonoscopy

Early detection and prompt treatment are essential in optimizing the treatment of colonic post-colonoscopy perforations. Patients presenting with such perforations should undergo immediate surgical intervention, which typically involves primary repair or resection (Recommendation 1B).

Recently, the frequency of colonic perforation has increased due to routinely performed advanced therapeutic endoscopy.

Over the last decade, many advancements have been streamlined to better address these perforations, yet there are no definitive guidelines for their optimal management [85].

Choosing a conservative or surgical approach depends on a variety of clinical factors [86].

Conservative management is typically used to treat patients in stable clinical condition without any signs of peritonitis. In published literature, fewer than 20% of patients with colonoscopy-related perforations were successfully treated with a non-surgical approach [87-89].

Although select patients may be responsive to non-operative therapy, most cases warrant prompt surgical intervention to minimize the extent of intraperitoneal contamination, thereby facilitating a single-step procedure that will likely reduce post-operative complications [88].

Further, timely intervention (shortened timeframe between perforation and treatment) results in improved patient outcome [90-92].

**An early laparoscopic approach is a safe and effective treatment for colonoscopy-related colonic perforation (Recommendation 1C)**.

Laparoscopic surgery is a prudent compromise that minimizes the risks of invasive surgery as well as those of insufficiently aggressive non-operative therapy [93,94].

If the area of perforation cannot be localized laparoscopically, the surgeon should begin with a laparotomy before proceeding further [95].

### Post-traumatic bowel injuries

The time between incidence and surgery is a significant determinant of morbidity in patients with injuries to visceral lumens (Hollow Viscus Injuries, HVIs). An expeditious diagnosis and prompt surgical intervention are recommended to improve the prognosis of patients presenting with HVIs (Recommendation 1C).

Hollow Viscus Injuries (HVIs) are associated with significant rates of morbidity and mortality. HVIs can occur by means of penetrating injury or blunt trauma, but they are less common in patients who have experienced blunt trauma than they are in those who have suffered a penetrating injury. In patients who have experienced blunt trauma, an accurate and timely diagnosis is often a difficult undertaking.

Several mechanisms of bowel injury have been documented in the wake of blunt abdominal trauma. The most common injury is the posterior crushing of the bowel segment between the seat belt and vertebra or pelvis. It results in local lacerations of the bowel wall, mural and mesenteric hematomas, transection of the bowel, localized devascularization, and full-thickness contusions. Devitalization of the areas of contusion may subsequently result in late perforation.

An important determinant of morbidity in patients with HVIs appears to be the interim time between injury and surgery. Only expeditious evaluation and prompt surgical action can improve the prognosis of these patients [96].

Older age, elevated Abdominal Abbreviated Injury Scores, significant extra-abdominal injuries, and delays exceeding 5 hours between admission and laparotomy were identified as significant risk factors predictive of patient mortality [97].

**Colonic non-destructive injuries should be primarily repaired. Although Delayed Anastomosis (DA) is suggested for patients with Destructive Colon Injuries (DCI) who must undergo a Damage Control Laparotomy (CDL), this strategy is not suggested for high risk patients (Recommendation 2C)**.

Management pathway of colonic injury has been evolving over last three decades. There has been general agreement that injury location does not affect the outcome.

Sharp and Coll. stratified 469 consecutive patients with full thickness penetrating colon injuries for 13 years by age, injury location and mechanism, and severity of shock.

314 (67%) patients underwent primary repair and 155 (33%) underwent resection. Most injuries involved the transverse colon (39%), followed by the ascending colon (26%), the descending colon (21%), and the sigmoid colon (14%). Overall, there were 13 suture line failures (3%) and 72 abscesses (15%). Most suture line failures involved injuries to the descending colon (p = 0.06), whereas most abscesses followed injuries to the ascending colon (p = 0.37). Injury location did not affect morbidity or mortality after penetrating colon injuries. For destructive injuries, operative decisions based on a defined algorithm rather than injury location achieved an acceptably low morbidity and mortality rate and simplifies management [98].

Colon injuries in the context of a Damage Control Laparotomy (DCL) are associated with high complication rates and an increased incidence of leakage [99]. Performing a Delayed Anastomosis (DA) during DCL for patients with Destructive Colon Injuries (DCI) who require surgical resection is an effective approach with complication rates comparable to those of conventional laparotomy and primary anastomosis and/or standard colostomy. However, in the event of extensive damage with vascular and visceral involvement, the surgical outcome depends largely on the damage control strategy. Hollow-organ injury following penetrating trauma should be transiently managed with suture ligation, staples, or simple suturing of the proximal and distal ends of the affected organ, while more definitive repairs (such as anastomosis, reconstruction, and colostomy) are typically deferred to later procedures [100-102]. Small bowel or colonic perforations are repaired with sutured closure. If the bowel requires resection and anastomosis, these steps are implemented at a later time and are not performed during initial management; this stepwise approach allows for better control of intestinal leakage without prolonging surgical time or increasing physiological stress. While the colostomy is a relatively quick procedure, it is not always recommended given that, during reanimation, the already edematous abdominal wall often swells to an even greater size, and the intestinal loop that is used to create the stoma may become necrotic due to hindered blood supply. Further, these circumstances can substantially prolong surgical time [100-102].

In 2011, Ordonez et al. performed a retrospective review of patients with penetrating DCI. The authors concluded that DAs should be performed for all patients presenting with DCI who undergo DCL; however, DAs are not recommended for patients with recurrent intra-abdominal abscesses, severe bowel wall edema and inflammation, or persistent metabolic acidosis. In these patients, a colostomy is a more appropriate alternative [103].

In 2011 Burlew et al. [104] reviewed patients requiring an open abdomen after trauma from January 1, 2002 to December 31, 2007. Type of bowel repair was stratified as immediate repair, immediate anastomosis, delayed anastomosis, stoma and a combination.

During the 6-year study period, 204 patients suffered enteric injuries and were managed with an open abdomen.

Enteric injuries were managed with immediate repair (58), immediate anastomosis (15), delayed anastomosis (96), stoma (10), and a combination (22); three patients died before definitive repair. Sixty-one patients suffered intra-abdominal complications: 35 (17%) abscesses, 15 (7%) leaks, and 11 (5%) enterocutaneous fistulas. The majority of patients with leaks had a delayed anastomosis. Leak rate increased as one progresses toward the left colon (small bowel anastomoses, 3% leak rate; right colon, 3%; transverse colon, 20%; left colon, 45%). There was a significant trend toward higher incidence of leak with closure day, with closure after day 5 having a four times higher likelihood of developing leak (3% vs. 12%, p = 0.02).

### Gastroduodenal perforation

Surgery is the treatment of choice for perforated peptic ulcers. In selected cases (patients younger than 70 years of age without septic shock or peritonitis and showing no spillage of water-soluble contrast medium in a gastroduodenogram), non-operative management may be appropriate. However, if there is no improvement of clinical condition within 24 hours of initial non-operative treatment, the patient should undergo surgery (Recommendation 1A).

Research has shown that surgery is the most effective means of source control in patients with peptic ulcer perforations [105-107].

Patients with perforated peptic ulcers may respond to conservative treatment without surgery. Such conservative treatment consists of nasogastric aspiration, antibiotics, and antisecretory therapy. However, patients older than 70 years of age with significant comorbidities, septic shock upon admission, and longstanding perforation (> 24 hours) are associated with higher mortality rates when non-operative treatment is attempted [107-109].

Delaying the time of surgery beyond 12 hours after the onset of clinical symptoms reduces the efficacy of the procedure, resulting in poorer patient outcome [110].

Simple closure with or without an omental patch is a safe and effective procedure to address small perforated ulcers (< 2 cm) (Recommendation 1A).

In the event of large perforated ulcers, concomitant bleeding or stricture, resectional gastroduodenal surgery may be required. Intraoperative assessment enables the surgeon to determine whether or not resection is the proper course of action (Recommendation 1B).

Different techniques for simple closure of perforations have been described and documented in detail.

In 2010, Lo et al. conducted a study to determine if an omental patch offers any clinical benefit that is not offered by simple closure alone [111].

The study demonstrated that, in terms of leakage rates and overall surgical outcome, covering the repaired perforated peptic ulcer with an omental patch did not convey additional advantages compared to simple closure alone. The authors of the investigation concluded that further prospective, randomized studies were needed to clarify the safety and feasibility of simple closure without the support of an omental patch.

In the event of a small perforated gastroduodenal peptic ulcer, no significant differences in immediate post-operative conditions were reported when comparing simple closure and surgery [106,111-115]

The role of resectional surgery in the treatment of perforated peptic gastroduodenal disease is poorly understood; many reports recommend gastrectomy only in select patients with large gastric perforations and concomitant bleeding or stricture [116-120].

Laparoscopic repair of perforated peptic ulcers can be a safe and effective procedure for experienced surgeons (Recommendation 1A).

Aside from reduced post-operative analgesic demands, the post-operative outcome of the laparoscopic approach does not differ significantly from that of open surgery. In all reported studies, patients presented with small ulcers and received simple sutures, and many also received an omental patch. There were no studies reporting emergency laparoscopic resection or laparoscopic repair of large ulcers [121-126].

When a pathologist is available, frozen sections should be prepared from biopsied tissue to better assess the nature of gastric perforations (Recommendation 2C).

If a patient has a curable tumor and is of a stable clinical condition (no septic shock, localized peritonitis, or other comorbidities) the ideal treatment is a gastrectomy (total or sub-total) with D2 lymph-node dissection. For patients with a curable tumor complicated by poor underlying conditions, a two-stage radical gastrectomy is recommended (first step: simple repair, second step: elective gastrectomy). By contrast, simple repair is recommended for patients in poor clinical condition with non-curable tumors (Recommendation 2C).

Surgery is the treatment of choice for cases of perforated gastric cancer. In most instances, gastric carcinoma is not suspected as the cause of perforation prior to an emergency laparotomy, and the diagnosis of malignancy is often made following intra- and post-operative examination. The treatment is intended to both manage the emergency condition of peritonitis and fulfil the technical demands of oncological intervention. Perforation alone does not significantly affect long-term survival rates following gastrectomies [127]; similarly, differed resections (i.e. two-stage radical gastrectomy) do not typically affect long-term recovery [128,129].

The presence of pre-operative shock appears to be the most significant prognostic factor adversely affecting post-operative survival rates following surgery for perforated gastric cancer [130].

Even in the presence of concurrent peritonitis, patients with perforated gastric cancer should undergo gastric resection; the only exception to this recommendation occurs when a patient is hemodynamically unstable or has unresectable cancer [131-133].

Early detection and prompt treatment are essential in optimizing the management of patients with post-Endoscopic Retrograde Cholangiopancreatography (ERCP) duodenal perforation.

Stable patients may be managed non-operatively. The timing of surgery following failed conservative treatment greatly influences the outcome of patients with post-ERCP duodenal perforation (Recommendation 2C).

The use of ERCP has transitioned from a diagnostic tool to a primarily therapeutic intervention in the treatment of pancreaticobiliary disorders. Several studies [134-137] have reported an elevated rate of ERCP-related perforation, increasing from 0.3% to 1.0%. Duodenal perforations may be retroperitoneal (typically in the periampullary region following sphincterotomy) or intraperitoneal (typically in the lateral wall following adjacent endoscope passage). Intraperitoneal perforations are often large, and affected patients may require immediate surgery [138].

Diagnoses of nosocomial, procedure-related perforations are made by evaluating clinical findings, particularly radiographic imaging with contrast examinations (preferably CT). The presence of retroperitoneal air upon CT analysis does not linearly correlate with the severity of the condition or the need for surgery [139,140].

If there is any suspicion of perforation, the surgeon must promptly diagnose the patient and immediately initiate systemic support, including broad-spectrum antibiotics and intravenous resuscitation. Following clinical and radiographic examination, the mechanism, site, and extent of injury should be taken into account when selecting a conservative or surgical approach [141].

Despite extensive retroperitoneal air observed in CT analysis, successful non-operative management of sphincterotomy-related retroperitoneal perforations is possible, provided that the patient remains stable [142,143]. In contrast, if a patient develops abdominal pain, becomes febrile, or appears critically ill, surgical exploration should be considered for repair or drainage, especially in the case of elderly or chronically ill patients who are less able to withstand physiological stress.

Early surgical intervention often facilitates ensuing primary repair strategies, similar in principle to closure of duodenal perforations secondary to duodenal ulcers. Delayed repair following failed non-operative treatment can be devastating and may require duodenal diversion and drainage without repair of the actual perforation.

Several novel methods of managing ERCP-induced perforation have been reported in recent literature [143,144]. Some patients have been managed successfully with an endoclipping device; however, this procedure is somewhat precarious given that adequate closure requires inclusion of the submucosal layer of the bowel wall, which clips cannot reliably ensure. Patients must be carefully selected for this procedure; the clipping method is only appropriate for patients who meet the criteria for conservative management (such as the absence of peritoneal signs) and who present with small, well-defined perforations detected without delay. The majority of pancreaticobiliary and duodenal perforations (70%) secondary to periampullary endoscopic interventions can be treated non-operatively [144] by means of nasogastric drainage, antibiotic coverage and nutritional support.

### Small bowel perforations

Jejunoileal perforations are a relatively uncommon source of peritonitis in Western countries compared to less developed regions where such intestinal perforations are a frequent contributor to high morbidity and mortality rates [145,146].

Although prompt surgery correlates with better clinical outcomes, there is widespread disagreement among the medical community regarding the proper surgical course of action; surgeons advocate a wide array of procedures, including simple closure, wedge excision or segmental resection and anastomosis, ileostomy, and side-to-side ileo-transverse anastomosis following primary perforation repair.

**Surgery is the treatment of choice for patients with small bowel perforations (Recommendation 1A)**.

In the event of small perforations, primary repair is recommended. However, when resection is required, subsequent anastomosis has not been shown to reduce post-operative morbidity and mortality rates. (Recommendation 2B).

**Further, only treatment centers with surgeons who are experienced in laparoscopic procedures should utilize the laparoscopic approach (Recommendation 2C)**.

Primary repair of perforated bowels is preferable to resection and anastomosis due to lower complication rates, although it should be noted that the optimal outcome in these cases may be attributable to the limited tissue injury of minor perforations [145,146].

Patients with malignant lesions, necrotic bowels, perforations associated with mesenteric vascular injuries, or multiple contiguous perforations should not undergo primary repair [147].

During resection, the entire diseased segment is excised, leaving healthy, well perfused ends for anastomosis. The technique used for the enteroenterostomy (stapled or hand-sewn) seems to have little impact on the anastomotic complication rate.

Primary bowel anastomosis must be approached cautiously in the presence of gross purulent or feculent peritonitis due to high rates of serious complications [146].

While laparoscopic management of small bowel perforations was extensively reported in published literature, there were no studies comparing laparoscopy to open surgery [147].

Among small bowel perforations, typhoid ileal perforation remains a serious complication of typhoid enteritis in many tropical countries, with mortality rates as high as 20-40% [148]. Furthermore, the increased incidence of *S. typhi* infections in patients with Acquired Immunodeficiency Syndrome (AIDS) raises the possibility of resurgent typhoid fever in the developed world [149].

No meta-analyses have been published on the subject of typhoid ileal perforation. In a recent prospective study, 53 consecutive patients with typhoid perforation were surgically treated; the morbidity rate for this series of procedures was 49.1%, and the most common post-operative complications included wound infection, wound dehiscence, burst abdomen, residual intra-abdominal abscesses, and enterocutaneous fistulae. The mortality rate was 15.1% and was significantly affected by the presence of multiple perforations, severe peritoneal contamination, and burst abdomen (p value < 0.05, odds ratio > 1) [150].

The morbidity and mortality rates do not depend on the surgical technique, but rather on the general status of the patient, the virulence of the pathogens, and the duration and character of disease evolution preceding surgical treatment. It is therefore important to provide attentive pre-operative management, including aggressive resuscitation by means of intravenous hydration and adequate antibiotic coverage. During surgery, thorough abdominal lavage is important in cases of serious abdominal suppuration [151]. Surgical treatment includes simple closure of the perforation, ileal resection, and side-to-side ileo-transverse colostomy or diverting ileostomy [148,152,153].

Primary repair should be performed for patients with minor symptoms and with perioperative findings of minimal fecal contamination of the peritoneal cavity. In the event of enteric perforation, early repair is typically more effective than a temporary ileostomy given that repair is more cost effective and is free of ileostomy-related complications. However, in delayed cases, there can be severe inflammation and edema of the bowel, resulting in friable tissue that complicates handling and suturing of the bowel. Primary closure of the perforation is therefore likely to leak, which is the etiological basis of the high incidence of fecal peritonitis and fecal fistulae associated with the procedure. Surgeons should perform a protective ileostomy to address fecal peritonitis and reduce mortality rates in the immediate term. The ileostomy serves to divert, decompress, and exteriorize, and in doing so, appears to have lower overall morbidity and mortality rates than other surgical procedures. The ileostomy is particularly useful for patients in critical condition presenting late in the course of illness when it often proves to be a life saving procedure.

### Acute cholecystitis

A laparoscopic cholecystectomy is a safe and effective treatment for acute cholecystitis. (Recommendation 1A).

The laparoscopic versus open cholecystectomy debate has been extensively investigated. Beginning in the early 1990s, techniques for laparoscopic treatment of the acutely inflamed gallbladder were streamlined and today the laparoscopic cholecystectomy is employed worldwide to treat acute cholecystitis.

Many prospective trials have demonstrated that the laparoscopic cholecystectomy is a safe and effective treatment for acute cholecystitis [154-158].

An early laparoscopic cholecystectomy is a safe treatment for acute cholecystitis and generally results in shorter recovery time and hospitalization compared to delayed laparoscopic cholecystectomies. (Recommendation 1A).

Timing is perhaps the most important factor in the surgical treatment of acute gallstone cholecystitis (AGC).

Evidence from published literature [159-162] demonstrates that, compared to delayed laparoscopic cholecystectomies, early laparoscopic cholecystectomies performed to treat acute cholecystitis reduce both recurrence rates and the overall length of hospital stay. A promptly performed laparoscopic cholecystectomy is therefore the most cost-effective means of treating acute cholecystitis.

In recent years, the medical community has debated the possible risk factors predictive of perioperative conversion to an open cholecystectomy from a laparoscopic approach in cases of acute cholecystitis [163,164].

Systematic evaluation of risk factors for laparoscopic to open conversion during cholecystectomies in patients with acute cholecystitis may help predict procedural difficulties and optimize surgical strategies of high-risk cases.

A delayed laparoscopic cholecystectomy is perhaps the most significant risk factor predictive of eventual laparoscopic to open conversion during a cholecystectomy in cases of acute cholecystitis [165].

In 2011, researchers published an analysis of patients undergoing urgent laparoscopic cholecystectomies (LCs) for acute cholecystitis based on the prospective database of the Swiss Association of Laparoscopic and Thoracoscopic Surgery [166]. The patients were grouped according to the time lapsed between hospital admission and laparoscopic cholecystectomy (admission day: d0, subsequent days of hospitalization: d1, d2, d3, d4/5, d ≥ 6). Delaying LC resulted in the following shifts in patient outcome: significantly higher conversion rates (increasing from 11.9% at d0 to 27.9% at d ≥ 6, P < 0.001), increased postoperative complications (increasing from 5.7% to 13%, P < 0.001), elevated repeat operation rates (increasing from 0.9% to 3%, P = 0.007), and significantly longer postoperative hospitalization (P < 0.001).

**Percutaneous cholecystostomy can be used to safely and effectively treat acute cholecystitis patients who are ineligible for open surgery. Whenever possible, percutaneous cholecystostomies should be followed by laparoscopic cholecystectomies (Recommendation 2C)**.

No randomized studies have been published that compare the clinical outcomes of percutaneous and traditional cholecystostomies. It is not currently possible to make definitive recommendations regarding percutaneous cholecystostomies (PC) or traditional cholecystectomies in elderly or critically ill patients with acute cholecystitis.

Whenever possible, percutaneous cholecystostomies should be followed by laparoscopic cholecystectomies.

A literature database search was performed on the subject of percutaneous cholecystostomies in the elderly population [167].

Successful intervention was observed in 85.6% of patients with acute cholecystitis. A total of 40% of the patients treated with PC were later cholecystectomized, resulting in a mortality rate of 1.96%. The overall mortality rate of the procedure was 0.36%, but 30-day mortality rates were 15.4% in patients treated with PC and 4.5% in those treated with a traditional cholecystectomy (P < 0.001).

Recently, several studies have confirmed the effects of cholecystostomies in critically ill patients [168], elderly patients [169], and surgically high-risk patients [170-174].

**Early diagnosis of gallbladder perforation and immediate surgical intervention may substantially decrease morbidity and mortality rates (Recommendation 1C)**.

Gallbladder perforation is an unusual form of gallbladder disease. Early diagnosis of gallbladder perforation and immediate surgical intervention are of utmost importance in decreasing morbidity and mortality rates associated with this condition.

Perforation is rarely diagnosed pre-operatively. Delayed surgical intervention is associated with elevated morbidity and mortality rates, increased likelihood of ICU admission, and prolonged post-operative hospitalization [175-179].

### Ascending cholangitis

Ascending cholangitis is a life-threatening condition that must be treated in a timely manner.

Early treatment, which includes appropriate antibiotic coverage, hydratation, and biliary decompression, is of utmost importance in the management of acute cholangitis (Recommendation 1A).

The appropriatness of biliary drainage in patients with acute cholangitis depends on specific clinical findings, and this procedure may be secondary to a previous failed treatment.

Cholangitis varies greatly in severity, ranging from a mild form requiring parenteral antibiotics to severe or suppurative cholangitis, which requires early drainage of the biliary tree to prevent further complications [180].

Retrospective studies have shown that, 20–30 years ago, when biliary drainage was not available, the mortality rate of conservatively treated acute cholangitis was extremely high [181].

Given that emergency biliary drainage in patients with acute cholangitis is not always necessary or feasible, it is very important that surgeons promptly and effectively triage patients, distinguishing those who require this urgent procedure from those who do not.

In 2001, Hui et al. [182] published a prospective study investigating predictive criteria for emergency biliary decompression for 142 patients with acute cholangitis. Emergency ERCP was associated with fever, a maximum heart rate exceeding 100 beats per minute, albumin less than 30 g/L, bilirubin greater than 50 μmol/L, and prothrombin time exceeding 14 seconds.

There are 3 common methods used to perform biliary drainage: endoscopic drainage, percutaneous transhepatic drainage, and open drainage.

Endoscopic drainage of the biliary tree is safer and more effective than open drainage (Recommendation A).

Endoscopic biliary drainage is a well-established means of biliary decompression for patients with acute cholangitis caused by malignant or benign biliary disease and associated biliary obstruction [183,184].

Many retrospective case-series studies have also demonstrated the efficacy of percutaneous transhepatic drainage.

Endoscopic modalities of biliary drainage are currently favored over percutaneous procedures due to reduced complication rates. There are currently no RCTs comparing endoscopic and percutaneous drainage. (Recommendation 2C).

Currently, only retrospective studies have been published comparing the safety and effectiveness of endoscopic and percutaneous transhepatic biliary drainage in the treatment of acute obstructive suppurative cholangitis. These reports confirmed the clinical efficacy of endoscopic drainage as well as its ability to facilitate subsequent endoscopic or surgical intervention [185].

Open drainage should only be performed for patients for whom endoscopic or percutaneous transhepatic drainage has failed or is otherwise contraindicated (Recommendation 2C).

Given the shortened length of hospitalization and the rarity of serious complications such as intraperitoneal hemorrhage and biliary peritonitis, endoscopic drainage is preferred to open drainage [186-189].

### Post-operative intra-abdominal infections

Post-operative peritonitis can be a life-threatening complication of abdominal surgery associated with high rates of organ failure and mortality. Treating patients with post-operative peritonitis requires supportive therapy of organ dysfunction, source control of infection via surgery and/or drainage, and intensive antimicrobial therapy [190].

Treatment recommendations are of little value given that randomized clinical trials are extremely difficult to perform for this particular pathology, and consequently, little relevant literature is available on the subject.

Percutaneous drainage is the optimal means of treating post-operative localized intra-abdominal abscesses when there are no signs of generalized peritonitis (Recommendation 2C).

Several retrospective studies in the fields of surgery and radiology have documented the effectiveness of percutaneous drainage in the treatment of post-operative localized intra-abdominal abscesses [191-193].

Source control should be initiated as promptly as possible following detection and diagnosis of post-operative intra-abdominal peritonitis. Ineffective control of the septic source is associated with significantly elevated mortality rates (Recommendation 1C).

Inability to control the septic source is associated with significant increases in patient mortality.

Organ failure and/or subsequent re-laparotomies that have been delayed for more than 24 hours both result in higher rates of mortality for patients affected by post-operative intra-abdominal infections [194].

Physical and laboratory tests are of limited value in diagnosing abdominal sepsis. CT scans typically offer the greatest diagnostic accuracy. Early re-laparotomies appear to be the most effective means of treating post-operative peritonitis [195].

### Re-laparotomy strategy

In certain instances infection can lead to an excessive immune response and sepsis may progress to severe sepsis, septic shock, or multiple organ dysfunction syndrome (MODS).

In these cases, patients are severely destabilized by the septic shock and will likely experience increased complication and mortality rates [196].

These patients benefit from aggressive surgical treatment, prompt intervention, and successive follow-up surgeries (“re-operations”) to better control MODS triggered by the ongoing intra-abdominal infection [197].

Deciding if and when to perform a re-laparotomy in cases of secondary peritonitis is largely subjective and based on professional experience. Factors indicative of progressive or persistent organ failure during early post-operative follow-up analysis are the best indicators of ongoing infection [198].

Three methods are currently employed for local mechanical management of abdominal sepsis following an initial laparotomy:

(1) Open abdomen

(2) Planned re-laparotomy

(3) On-demand re-laparotomy

Given the procedure’s ability to streamline healthcare resources, reduce overall medical costs, and prevent the need for further re-laparotomies, the on-demand re-laparotomy is recommended for patients with severe peritonitis. (Recommendation 1A).

In 2007, van Ruler et al. [199] published a randomized, clinical study comparing planned and on-demand re-laparotomy strategies for patients with severe peritonitis.

In this trial, a total of 232 patients with severe intra-abdominal infections were randomized (116 planned and 116 on-demand).

In the planned re-laparotomy group, procedures were performed every 36 to 48 hours following the index laparotomy to inspect, drain, lavage, and perform other necessary abdominal interventions to address residual peritonitis or new infectious focuses.

In the on-demand re-laparotomy group, procedures were only performed for patients who demonstrated clinical deterioration or lack of improvement that was likely attributable to persistent intra-abdominal pathology.

Patients in the on-demand re-laparotomy group did not exhibit a significantly lower rate of adverse outcomes compared to patients in the planned re-laparotomy group, but they did show a substantial reduction in subsequent re-laparotomies and overall healthcare costs.

The on-demand group featured a shorter median ICU stay (7 days for on-demand group < 11 days for planned group; P = 0.001) and a shorter median length of hospitalization (27 days for on-demand group < 35 days for planned group; P = 0.008). Direct per-patient medical costs were reduced by 23% using the on-demand approach.

Members of our Expert Panel emphasize, however, that an on-demand strategy is not a forgone conclusion for all patients presenting with severe secondary peritonitis; that is, secondary peritonitis alone isn’t necessary and sufficient to automatically preclude other alternatives. The decision to implement an on-demand strategy is based on contextual criteria and should be determined on a case-by-case basis.

For “wait-and-see” management of on-demand patients requiring follow-up surgery, early re-laparotomies appear to be the most effective means of treating post-operative peritonitis and controlling the septic source [200-202].

Organ failure and/or subsequent re-laparotomies delayed for more than 24 hours both correlate with higher mortality rates for patients affected by post-operative intra-abdominal infections [203].

Deciding whether or not to perform additional surgeries is context sensitive and depends on the surgeon and on his or her professional experience; no telltale clinical parameters are available [204,205].

The findings of a single RTC are hardly concrete, and further studies are therefore required to better define the optimal re-laparotomy strategy.

The open abdomen remains a viable option for treating intra-abdominal sepsis. The benefits of maintaining an open abdomen include ease of subsequent exploration, control of abdominal contents, reduced risk of intra-abdominal hypertension and abdominal compartment syndrome, and fascial preservation to ensure proper closure of the abdominal wall. However, prolonged exposure of abdominal viscera can result in additional complications, including infection, sepsis, and fistula formation (Recommendation 1C).

The open abdomen is the most technically straightforward means of conducting a planned follow-up procedure.

Open treatment was first used to manage severe intra-abdominal infections and pancreatic necrosis [200].

However, severe complications such as evisceration, fistula formation, and the development of giant incisional hernias were frequently observed in this procedure.

Temporary closure of the abdomen may be achieved by using gauze and large, impermeable, self-adhesive membrane dressings, both absorbable and non-absorbable meshes, and negative pressure therapy devices.

At present, negative pressure techniques (NPT) have become the most extensively employed means of temporary closure of the abdominal wall.

In recent years, open abdomen procedures have increased dramatically due to streamlined “damage control” techniques in life-threatening conditions, recognition and treatment of intra-abdominal hypertension and abdominal compartment syndrome, and important clinical findings regarding the management of severe intra-abdominal sepsis.

A more comprehensive understanding of the pathophysiology of open abdomen conditions as well as the development of new technologies for temporary abdominal wall closure have improved the management and outcome of patients undergoing this procedure [203].

Severe intra-abdominal infection is a progressive condition; affected patients progress from sepsis to severe sepsis with organ dysfunction and ultimately to septic shock.

This stepwise progression is characterized by excessive proinflammation, which causes vasodilation, hypotension, and myocardial depression. These effects combined with endothelial activation and Diffused Intravascular Coagulopathy (DIC), cause ongoing endothelial leakage, cellular shock, and microvascular thrombosis. Outwardly, clinical manifestations are characterized by septic shock and progressive MOF. In this situation, a surgeon must decide whether or not to perform a “damage control” laparotomy, thereby providing prompt and aggressive source control to curb the momentum of crescendoing sepsis.

Advantages of the open abdomen include prevention of abdominal compartment syndrome (ACS). In the event of septic shock, massive fluid resuscitation, bowel edema and forced closure of a non-compliant abdominal wall all contribute to intra-abdominal hypertension (IAH). Elevated intra-abdominal pressure (IAP) adversely affects the physiological processes of pulmonary, cardiovascular, renal, splanchnic, and central nervous systems. The combination of IAH and other physiological stressors contributes to significantly elevated morbidity and mortality rates.

Several studies have investigated open abdomen in the context of intra-abdominal infections, generating great interest and optimism in the medical community [206-209].

However, in 2007 a randomized study compared open and closed “on-demand” management of severe peritonitis. The study was terminated following the inclusion of only 40 patients after acknowledging the clearly discernable clinical disadvantages of the open abdomen group (55% and 30% mortality rates for open and closed procedures, respectively). It should be noted that, in this study, the open abdomen was managed exclusively with non-absorbable polypropylene mesh and without negative pressure therapy [210].

Following stabilization of the patient, surgeons should attempt early, definitive closure of the abdomen. Primary fascial closure may be possible when there is minimal risk of excessive tension or recurrence of IAH (Recommendation 1C).

When early, definitive fascial closure is not possible, progressive closure should be attempted each time the patient returns for subsequent procedures.

For patients with persistent large fascial defects, it is suggested that surgeons implement bridging with biological materials (Recommendation 1C).

Following stabilization of the patient, the primary objective is early and definitive closure of the abdomen to minimize complications associated with OA [206].

For many patients, primary fascial closure may be possible within a few days of the initial operation [206].

In other patients, early definitive fascial closure may not be possible. In these cases, surgeons should attempt progressive closure, in which the abdomen is incrimentally closed each time the patient undergoes a subsequent surgery.

Many methods of fascial closure have been described in medical literature [211-216]. In many cases abdominal closure is only partially achieved, resulting in large, debilitating hernias of the abdominal wall that will eventually require complex surgical repair. In these cases, bridging with biological mesh is recommended [217].

### Antimicrobial therapy

Initial antibiotic therapy for IAIs is typically empirical in nature because the patient needs immediate attention, and microbiological data (culture and susceptibility results) can require up to 48 hours before they are available for a more detailed analysis.

IAIs can be treated with either single or multiple antimicrobial regimens depending on the range requirements of antimicrobial coverage.

Beta-lactam/beta-lactamase inhibitor combinations exhibit in vitro activity against gram-positive, gram-negative, and anaerobic organisms [218,219] and are viable options for empirical treatment of IAIs [218]. However, the increasing prevalence of drug-resistant Enterobacteriaceae observed in community-acquired infections restricts this regimen’s empirical use to patients who are not at risk for these drug-resistant microorganisms [220].

In the past, Cephalosporins have often been used in the treatment of intra-abdominal infections. Among third generation cephalosporins, subgroups with both limited and strong activity against *Pseudomonas aeruginosa* (cefepime and ceftazidime) have been used in conjunction with metronidazole to treat IAIs. Enterobacteriaceae can have acquired resistance to both cephalosporins, while such resistance is intrinsic in Enterococci [221-223].

In light of the increasing prevalence of ESBL-producing enterobacteriaceae due to selection pressures related to overuse of cephalosporins, routine use of these antibiotics is strongly discouraged.

Aztreonam is a parenteral synthetic beta-lactam antibiotic and the first monobactam marketed for clinical use. The drug exhibits potent in vitro activity against a wide spectrum of gram-negative aerobic pathogens (including *Pseudomonas aeruginosa*), but its routine use is discouraged due to selection pressures favoring resistant strains, and it therefore shares the same constraints associated with cephalosporin use.

Carbapenems offer a wide spectrum of antimicrobial activity against gram-positive and gram-negative aerobic and anaerobic pathogens (with the exception of MDR resistant gram-positive cocci). For more than 2 decades, carbapenems have been considered the agents of “last resort” for multidrug-resistant infections caused by Enterobacteriaceae. In the last decade, increased carbapenem consumption has been associated with an increased emergence of carbapenem resistance among Enterobacteriacea, particularly in *Klebsiella pneumoniae*.

The recent and rapid spread of serine carbapenemases in *Klebsiella pneumoniae* (known as Klebsiella pneumoniae carbapenemases or KPCs) has become an issue of crucial importantance in hospitals worldwide [224].

Group 1 carbapenems include ertapenem, a once-a-day carbapenem that shares the activity of imipenem and meropenem against most species, including extended-spectrum beta-lactamase (ESBL)-producing pathogens, but is not active against Pseudomonas and Enterococcus species [225,226].

Group 2 includes imipenem/cilastatin, meropenem, and doripenem, which share activity against non-fermentative gram-negative bacilli. Researchers have reported doripenem’s slightly elevated in vitro activity against certain Pseudomonas strains in registrative trials [227].

Further, given their excellent tissue penetration and strong activity against aerobic gram-negative bacteria, fluoroquinolones have been widely used in recent years for treatment of IAIs. It should also be noted that all fluoroquinolones are rapidly and almost completely absorbed from the gastrointestinal tract.

A combination of ciprofloxacin/metronidazole has been perhaps the most commonly used regimen for complicated IAIs in recent years. The latest quinolone, Moxifloxacin, has demonstrated to be active against a wide range of aerobic gram-positive and gram-negative species [228]. Compared to ciprofloxacin, moxifloxacin has enhanced activity against gram-positive bacteria and decreased activity against gram-negative bacteria [229]. Among quinolones, moxifloxacin appears to also be effective against *Bacterioides fragilis*, suggesting that the drug may be equally effective without co-administered antianaerobic agents [230-232]. However, in recent years, the ever-increasing incidence of drug resistance among Enterobacteriaceae and non-fermentative gram-negative bacilli has discouraged the drug’s use in empirical regimens.

Aminoglycosides are particularly active against aerobic gram-negative bacteria and act synergistically against certain gram-positive organisms. They are effective against *Pseudomonas aeruginosa* but are ineffective against anaerobic bacteria. Aminoglycosides may be suboptimal for treatment of abscesses or intra-abdominal infections due to their low penetration in acidic environments [233].

Tigecycline is a parenteral glycylcycline antibiotic derived from minocycline. It is the first representative of the glycylcycline class of antibacterial agents to be marketed for clinical use [234,235].

While tigecycline does not feature in vitro activity against *P. aeruginosa* or *P. mirabilis,* it remains a viable treatment option for complicated IAIs due to its favorable in vitro activity against anaerobic organisms, Enterococci, several ESBL- and carbapenemase-producing Enterobacteriaceae, Acinetobacter species, and *Stenotrophomonas maltophilia*[236-238].

The use of tigecycline to treat IAIs is particularly useful in light of its unique pharmacokinetic properties; the drug is eliminated by active biliary secretion and is therefore able to establish high biliary and fecal concentrations [239].

Cultures from the site of infection are always recommended for patients with healthcare-associated infections or with community-acquired infections at risk for resistant pathogens. In these patients, the causative pathogens and the related resistance patterns are not readily predictable and therefore require further analysis (Recommendation 1C).

The results of microbiological analysis are helpful in designing therapeutic strategies for individual patients to customize antibiotic treatments and ensure adequate antimicrobial coverage.

Although it has been documented that bacteriological cultures have little impact on the course of treatment of common conditions like appendicitis [240], in this era of prevalent drug-resistant microorganisms involved in both nosocomial and community-acquired infections, the threat of resistance is a source of major concern that cannot be ignored.

In 2010, a review was published investigating the value of peritoneal fluid cultures in cases of appendicitis [241]. All included studies focusing on the use of intra-peritoneal swabs were open, non-randomized, and retrospective; further, they featured incompletely matched control groups and non-standardized swab collection techniques, and therefore offered limited statistical power with which to suggest modifications of surgical practice. Until controlled trial data of more reliable methodological quality become available, clinicians should continue the use of peritoneal swabs, especially for high-risk patients.

Cultures should be taken from intra-abdominal samples during surgical or interventional drainage procedures. Surgeons must ensure sufficient volume (a minimum of 1 mL of fluid or tissue) before sending the samples to a clinical laboratory by means of a transport system that properly handles the samples so as not to damage them or compromise their integrity.

The empirically designed antimicrobial regimen depends on the underlying severity of infection, the pathogens presumed to be involved, and the risk factors indicative of major resistance patterns (Recommendation 1B).

Predicting the pathogens and potential resistance patterns of a given infection begins by establishing whether the infection is community-acquired or healthcare-associated (nosocomial).

The major pathogens involved in community-acquired intra-abdominal infections are Enterobacteriaceae, Streptococcus species, and anaerobes (especially *B. fragilis*).

Contrastingly, the spectrum of microorganisms involved in nosocomial infections is significantly broader. In the past 20 years, the incidence of healthcare-associated infections caused by drug-resistant microorganisms has risen dramatically, probably in correlation with escalating levels of antibiotic exposure and increasing frequency of patients with one or more predisposing conditions, including elevated severity of illness, advanced age, degree of organ dysfunction, low albumin levels, poor nutritional status, immunodepression, presence of malignancy, and other comorbidities.

Although the transmission of multidrug-resistant organisms is most frequently observed in acute care facilities, all healthcare settings are affected by the emergence of drug-resistant pathogens.

In past decades, an increased prevalence of infections caused by antibiotic-resistant pathogens, including methicillin-resistant *Staphylococcus aureus*, vancomycin-resistant Enterococcus species, carbapenem-resistant *Pseudomonas aeruginosa*, extended-spectrum beta-lactamase (ESBL)-producing *Escherichia coli* and Klebsiella species, multidrug-resistant Acinetobacter species, and Candida species has been observed, particularly in cases of intra-abdominal infection [242-244].

For patients with severe sepsis or septic shock, early and properly administered empirical antimicrobial therapy can have a significant impact on the outcome, independent of the anatomical site of infection [245].

These data confirm the results of Riché et al. whose prospective observational study involving 180 consecutive patients with secondary generalized peritonitis demonstrated a significantly higher mortality rate for patients in septic shock (35% and 8% for patients with and without shock, respectively) [246].

International guidelines for the management of severe sepsis and septic shock (the Surviving Sepsis Campaign) recommend intravenously administered antibiotics within the first hour of onset of severe sepsis and septic shock and the use of broad-spectrum agents with adequate penetration of the presumed site of infection. Additionally, the employed antimicrobial regimen should be reassessed daily in order to optimize efficacy, prevent toxicity, minimize cost, and reduce selection pressures favoring resistant strains [10].

To ensure timely and effective administration of antimicrobial therapy for critically ill patients, clinicians must consider the pathophysiological and immunological status of the patient as well as the pharmacokinetic properties of the employed antibiotics (Recommendation 1C).

In the event of abdominal sepsis, clinicians must be aware that drug pharmacokinetics may be altered significantly in critically ill patients due to the pathophysiology of sepsis. The “dilution effect,” also known as the “third spacing phenomenon,” is very important for hydrophilic agents. Higher than standard loading doses of hydrophilic agents such as beta-lactams, aminoglycosides, and glycopeptides should be administered to ensure optimal exposure at the infection site, maintaining a therapeutic threshold that withstands the effects of renal function [247].

For lipophilic antibiotics such as fluoroquinolones and tetracyclines, the “dilution effect” in extracellular fluids may be mitigated during severe sepsis by the rapid redistribution of drugs to the interstitium from the intracellular compartment. Unlike observations of subtherapeutic administration of standard-dose hydrophilic antimicrobials, standard dosages of lipophilic antimicrobials are often sufficient to ensure adequate loading, even in patients with severe sepsis or septic shock [248].

Once initial loading is achieved, it is recommended that clinicians reassess the antimicrobial regimen daily, given that pathophysiological changes may occur that significantly alter drug disposition in critically ill patients.

Lower-than-standard dosages of renally excreted drugs must be administered in the presence of impaired renal function, while higher-than-standard dosages of renally excreted drugs may be required for optimal exposure in patients with glomerular hyperfiltration [249].

Table 2 overviews recommended dosing regimens of the most commonly used renally excreted antimicrobials.

**Table 2:** **Recommended dosing regimens (according to renal function) of the most commonly used renally excreted antimicrobials [**[248]**]**

	**Renal function**			
**Antibiotic**	**Increased**	**Normal**	**Moderately impaired**	**Severely impaired**
*Piperacillin/tazobatam*	16/2 g q24 h CI or 3.375 q6 h EI over 4 hours	4/0.5 g q6 h	3/0.375 g q6 h	2/0.25 g q6 h
*Imipenem*	500 mg q4 h or 250 mg q3 h over 3 hours CI	500 mg q6 h	250 mg q6 h	250 mg q12 h
*Meropenem*	1 g q6 h over 6 hours CI	500 mg q6 h	250 mg q6 h	250 mg q12 h
*Ertapenem*	ND	1 g q24 h	1 g q24 h	500 mg q24 h
*Gentamycin*	9 to 10 mg/kg q24 h^b^	7 mg/kg q24 h	7 mg/kg q36–48 h	7 mg/kg q48–96 h
*Amikacin*	20 mg/kg q24 h	15 mg/kg q24 h	15 mg/kg q36–48 h^b^	15 mg/kg q48–96 h
*Ciprofloxacin*	600 mg q12 h or 400 mg q8 h	400 mg q12 h	400 mg q12 h	400 mg q24 h
*Levofloxacin*	500 mg q12 h	750 mg q24 h	500 mg q24 h	500 mg q48 h
*Vancomycin*	30 mg/kg q24 h CI	500 mg q6 h	500 mg q12 h	500 mg q24–72 h
*Teicoplanin*	LD 12 mg/kg q12 h for 3 to 4 doses; MD 6 mg/kg q12 h	LD 12 mg/kg q12 h for 3 to 4 doses; MD 4 to 6 mg/kg q12 h	LD 12 mg/kg q12 h for 3 to 4 doses; MD 2 to 4 mg/kg q12 h	LD 12 mg/kg q12 h for 3 to 4 doses; MD 2 to 4 mg/kg q24 h
*Tigecycline*	LD 100 mg; MD 50 mg q12 h	LD 100 mg; MD 50 mg q12 h	LD 100 mg; MD 50 mg q12 h	LD 100 mg; MD 50 mg q12 h

The therapeutic approach undertaken by clinicians must take into account the activity of employed antimicrobials.

Antibiotics such as quinolones, daptomycin, tigecycline, aminoglycosides, polienes, and echinocandins exhibit concentration-dependent activity; as such, the dose should be administered in a once-a-day manner (or with the lowest possible daily administrations) in order to achieve zenithal plasma levels [249].

Beta-lactams, glycopeptides, oxazolidinones, and azoles exhibit time-dependent activity and exert optimal bactericidal activity when drug concentrations are maintained above the Minimum Inhibitory Concentration (MIC).

The efficacy of time-dependent antibacterial agents in severely ill patients is related primarily to the maintenance of supra-inhibitory concentrations, and therefore multiple daily dosing may be appropriate.

For these drugs, continuous intravenous infusion ensures the highest steady-state concentration under the same dosage constraints and may therefore be the most effective means of maximizing pharmacodynamic exposure [250,251].

For patients with community-acquired intra-abdominal infections (CA-IAIs), agents with a narrower spectrum of activity are preferred. However, if CA-IAI patients have prior exposure to antibiotics or serious comorbidities requiring concurrent antibioitic therapy, anti-ESBL-producer converage may be warranted. By contrast, for patients with healthcare-associated infections, antimicrobial regimens with broader spectra of activity are preferred (Recommendation 1B).

In the context of intra-abdominal infections, the main resistance problem is posed by ESBL-producing Enterobacteriaceae, which are alarmingly prevalent in nosocomial infections and frequently observed in community-acquired infections, albeit to a lesser extent.

The Study for Monitoring Antimicrobial Resistance Trends (SMART) program monitors the activity of antibiotics against aerobic gram-negative intra-abdominal infections. Hawser et al. reported susceptibility levels of key intra-abdominal pathogens in Europe in 2008 and noted that the number of viable treatment options available for empirical treatment of intra-abdominal infections had fallen dramatically [252].

Although a variety of factors can increase the risk of selection for ESBL producers, the most significant risk factors include prior exposure to antibiotics (especially third generation cephalosporins) and comorbidities requiring concurrent antibiotic therapy.

In a study published by Ben-Ami et al., researchers evaluated risk factors for non-hospitalized patients that increased susceptibility to ESBL-producing infections; the study compiled data from 6 treatment centers in Europe, Asia, and North America [253].

A total of 983 patient-specific isolates were analyzed; 890 [90.5%] were *Escherichia coli*; 68 [6.9%] were Klebsiella species; and 25 [2.5%] were *Proteus mirabilis*. Overall, 339 [34.5%] of the observed isolates produced ESBLs. Significant risk factors identified by multivariate analysis included recent antibiotic exposure, residence in long-term care facilities, recent hospitalization, and advanced age greater than 65 years. Additionally, men appeared to be more prone to these infections than women.

However, 34% of the analyzed ESBL isolates were derived from patients with no recent healthcare exposure.

Bacteria producing Klebsiella pneumoniae carbapenemases (KPCs) are rapidly emerging as a major source of multidrug-resistant infections worldwide. The recent emergence of carbapenem resistance among Enterobacteriaceae poses a considerable threat to hospitalized patients.

In addition to hydrolyzing carbapenems, KPC-producing strains are often resistant to a variety of other antibiotics, and effective treatment of these versatile and resilient pathogens has therefore become an important challenge for clinicians in acute care settings [254].

KPC-producing bacteria have become commonplace in nosocomial infections, especially in patients with previous exposure to antibiotics [255].

Further, *Pseudomonas aeruginosa* and *Acinetobacter baumannii* have exhibited alarming rates of increased resistance to a variety of antibiotics in hospitals and healthcare facilities worldwide. Both species are intrinsically resistant to several drugs and could acquire additional resistances to other important antimicrobial agents [256].

Although no supportive data are currently available, *P. aeruginosa* coverage is only generally recommended for patients with nosocomial intra-abdominal infections, despite the fact that, in certain subpopulations, an inexplicably high prevalence of *Pseudomonas aeruginosa* has been documented in association with community-acquired appendicitis, which may complicate empirical antibiotic therapy [257].

Among multidrug-resistant gram-positive bacteria, Enterococci remain a considerable challenge.

Empirical coverage of Enterococci is not generally recommended for patients with community-acquired IAIs. Studies have demonstrated that coverage against Enterococci offers little therapeutic benefit for patients with community-acquired infections [258,259].

In the context of community-acquired IAIs, antimicrobial therapy for Enterococci should be considered for immunocompromised patients, patients with valvular heart disease or prosthetic materials, and critically ill patients for whom empirical antimicrobial therapy has a significant impact on clinical outcome.

Methicillin-resistant *Staphylococcus aureus* (MRSA) is another multidrug-resistant gram-positive nosocomial pathogen known to cause severe morbidity and mortality worldwide [260].

Although community-acquired MRSA has been reported in other settings, there are no studies that have systematically documented MRSA in community-acquired intra-abdominal infections.

Patients with nosocomial intra-abdominal infections should not be treated empirically for MRSA unless the patient has a history of infections by this organism or there is reason to believe that the infection is associated with MRSA.

Appendices 1, 2, 3, 4, 5, 6, 7, 8, 9, 10 list recommended antimicrobial regimens.

Empirical antifungal therapy for Candida species is recommended for patients with nosocomial infections and for critically ill patients with community-acquired infections. An echinocandin regimen is recommended for critically ill patients with nosocomial infections (Recommendation 1B).

Although the epidemiological profile of Candida species has not yet been defined in the context of nosocomial peritonitis, its presence is clinically significant and is usually associated with poor prognoses.

Empirical antifungal therapy for Candida species is typically not recommended for patients with community-acquired intra-abdominal infections, with the notable exceptions of patients recently exposed to broad-spectrum antimicrobials and immunocompromised patients (due to neutropenia or concurrent administration of immunosuppressive agents, such as glucocorticosteroids, chemotherapeutic agents, and immunomodulators) [261].

However, considering the high mortality rate of Candida-related peritonitis, and given the poor outcome that could result from inadequate antimicrobial therapy for critically ill patients, antifungal coverage is recommended for these patients

In 2006, Montravers et al. published a retrospective, case–control study involving critically ill patients admitted to 17 French intensive care units (ICUs) [262].

The study demonstrated an increased mortality rate in cases of nosocomial peritonitis in which fungal isolates had been identified (48% and 28% mortality rates for fungal peritonitis and control groups, respectively p < 0.01). Upper gastrointestinal tract sites and positive identification of Candida species were found to be independent variables predictive of mortality for patients with nosocomial peritonitis.

More recently, Montravers et al. published the results of a prospective, non-interventional study involving 271 adult ICU patients with invasive Candida infections who received systemic antifungal therapy; the authors reported a mortality rate of 38% in a prospective cohort of 93 patients admitted to the ICU with candidal peritonitis [261].

Given the results of these studies, the inclusion of an anticandidal drug in empirical regimens for nosocomial IAIs seems appropriate.

The recently published Pappas IDSA guidelines for the treatment of invasive candidiasis do not dedicate a specific section to candidal peritonitis [263]. However, the use of echinocandins is generally recommended as a first-line empirical treatment for critically ill patients, while fluconazole is typically recommended for less severe conditions.

Applying these trends to IAIs, the use of echinocandins is recommended as a first-line treatment in cases of severe nosocomial IAI.

Knowledge of mechanisms of secretion of antibiotics into bile is helpful in designing the optimal therapeutic regimen for patients with biliary-related intra-abdominal infections (Recommendation 1C).

The bacteria most often isolated in biliary infections are *Escherichia coli* and *Klebsiella pneumonia,* gram-negative aerobes,, as well as certain anaerobes, particularly *Bacteroides fragilis*. Given that the pathogenicity of Enterococci in biliary tract infections remains unclear, specific coverage against these microorganisms is not routinely advised [264-266].

The efficacy of antibiotics in the treatment of biliary infections depends largely on the therapeutic level of drug concentrations [267-271].

The medical community has debated the use of antimicrobials with effective biliary penetration to address biliary infections. However, no clinical or experimental evidence is available to support the recommendation of biliary-penetrative antimicrobials for these patients. Other important factors include the antimicrobial potency of individual compounds and the effect of bile on antibacterial activity [270].

If there are no signs of persistent leukocytosis or fever, antimicrobial therapy for intra-abdominal infections should be shortened for patients demonstrating a positive response to treatment (Recommendation 1C).

An antimicrobial-based approach involves both optimizing empirical therapy and curbing excessive antimicrobial use to minimize selective pressures favoring drug resistance [271].

Shortening the duration of antimicrobial therapy in the treatment of intra-abdominal infections is an important strategy for optimizing patient care and reducing the spread of antimicrobial resistance.

The optimal duration of antibiotic therapy for intra-abdominal infections has been extensively debated.

Shorter durations of therapy have proven to be as effective as longer durations for many common infections. A prospective, randomized, double-blind trial comparing 3- and ≥ 5-day ertapenem regimens in 111 patients with community-acquired intra-abdominal infections reported similar cure and eradication rates (93% vs. 90% and 95% vs. 94% for 3- and > 5-day regimens, respectively) [272].

Studies have demonstrated a low likelihood of infection recurrence or treatment failure when antimicrobial therapy is discontinued in patients with complicated intra-abdominal infection who no longer show signs of infection.

Lennard et al. [273] compared post-operative outcomes in 65 intra-abdominal sepsis patients with and without leukocytosis and fever at the conclusion of antimicrobial therapy. Intra-abdominal sepsis patients at risk for post-operative infection were those who were afebrile with persistent leukocytosis or those who remained febrile after the antibiotics were discontinued.

Hedrick et al. [274] retrospectively analyzed the relationship between the duration of antibiotic therapy and infectious complications (i.e., recurrent infection by the same organism or renewed infectious focus at the same anatomical site). In the study, 929 patients with intra-abdominal infections associated with fever or leukocytosis were categorized into quartiles on the basis of either the total duration of antibiotic therapy or the duration of treatment following resolution of fever and leukocytosis. Shorter courses of antibiotics were associated with comparable or fewer complications than prolonged therapy.

These results suggest that antimicrobial therapy to address intra-abdominal infections should be shortened for patients who demonstrate a positive response to treatment, show no signs of persistent leukocytosis or fever, and are able to resume an oral diet.

## Conclusions

Despite advances in diagnosis, surgery, and antimicrobial therapy, mortality rates associated with complicated intra-abdominal infections remain exceedingly high.

WSES guidelines represent a contribution on this debated topic by specialists worldwide.

## Appendix 1. Antimicrobial therapy for community-acquired extra-biliary IAIs in stable, non-critical patients presenting with no ESBL-associated risk factors (WSES recommendations)

Community-acquired extra-biliary IAIs

Stable, non-critical patients

No risk factors for ESBL

AMOXICILLIN/CLAVULANATE

Daily schedule: 2.2 g every 6 hours (2-hour infusion time)

OR (in the event of patients allergic to beta-lactams):

CIPROFLOXACIN

Daily schedule: 400 mg every 8 hours (30-minute infusion time)

+

METRONIDAZOLE

Daily schedule: 500 mg every 6 hours (1-hour infusion time)

## Appendix 2. Antimicrobial therapy for community-acquired extra-biliary IAIs in stable, non-critical patients presenting with ESBL-associated risk factors (WSES recommendations)

Community-acquired extra-biliary IAIs

Stable, non-critical patients

ESBL-associated risk factors

ERTAPENEM

Daily schedule: 1 g every 24 hours (2-hour infusion time)

OR

TIGECYCLINE

Daily schedule: 100 mg LD then 50 mg every 12 hours

## Appendix 3. Antimicrobial therapy for community-acquired extra-biliary IAIs in critically ill patients presenting with no ESBL-associated risk factors (WSES recommendations)

Community-acquired extra-biliary IAIs

Critically ill patients (≥ SEVERE SEPSIS)

No risk factors for ESBL

PIPERACILLIN/TAZOBACTAM

Daily schedule: 8/2 g LD then 16/4 g/day via continuous infusion or 4.5 g every 6 hours (4-hour infusion time)

## Appendix 4. Antimicrobial therapy for community-acquired extra-biliary IAIs in critically ill patients presenting with ESBL-associated risk factors (WSES recommendations)

Community-acquired IAIs

Critically ill patients (≥ SEVERE SEPSIS)

ESBL-associated risk factors

MEROPENEM

Daily schedule: 500 mg every 6 hours (6-hour infusion time)

OR

IMIPENEM

Daily schedule: 500 mg every 4 hours (3-hour infusion time)

+/−

FLUCONAZOLE

Daily schedule: 600 mg LD then 400 mg every 24 hours (2-hour infusion time)

## Appendix 5. Antimicrobial therapy for biliary IAI in stable, non-critical patients presenting with no ESBL-associated risk factors (WSES recommendations)

Community-acquired biliary IAIs

Stable, non-critical patients

No risk factors for ESBL

AMOXICILLIN/CLAVULANATE

Daily schedule: 2.2 g every 6 hours (2-hour infusion time)

OR (in the event of patients allergic to beta-lactams)

CIPROFLOXACIN

Daily schedule: 400 mg every 8 hours (30-minute infusion time)

+

METRONIDAZOLE

Daily schedule: 500 mg every 6 hours (1-hour infusion time)

## Appendix 6. Antimicrobial therapy for biliary IAIs in stable, non-critical patients presenting with ESBL-associated risk factors (WSES recommendations)

Community-acquired biliary IAIs

Stable, non-critical patients.

Risk factors for ESBL

TIGECYCLINE

Daily schedule: 100 mg LD then 50 mg every 12 hours (2-hour infusion time)

## Appendix 7. Antimicrobial therapy for biliary IAIs in critically ill patients presenting with no ESBL-associated risk factors (WSES recommendations)

Community-acquired biliary IAIs

Critically ill patients (≥ SEVERE SEPSIS)

No risk factors for ESBL

PIPERACILLIN/TAZOBACTAM

Daily schedule: 8/2 g LD then 16/4 g/day via continuous infusion or 4.5 g every 6 hours (4-hour infusion time)

## Appendix 8. Antimicrobial therapy for biliary IAIs in critically ill patients presenting with ESBL-associated risk factors (WSES recommendations)

Community-acquired biliary IAIs

Critically ill patients (SEVERE SEPSIS)

Risk factors for ESBL

PIPERACILLIN

Daily schedule: 8 g by LD then 16 g via continuous infusion or 4 g every 6 hours (4-hour infusion time)

+

TIGECYCLINE

Daily schedule: 100 mg LD then 50 mg every 12 hours (2-hour infusion time)

+/−

FLUCONAZOLE

Daily schedule: 600 mg LD then 400 mg every 24 hours (2-hour infusion time)

## Appendix 9. Antimicrobial therapy for nosocomial IAIs in stable, non-critical patients (WSES recommendations)

Hospital-acquired IAIs

Stable, non-critical patients (< SEVERE SEPSIS)

Risk factors for MDR pathogens

PIPERACILLIN

Daily schedule: 8 g by LD then 16 g via continuous infusion or 4 g every 6 hours (4-hour infusion time)

+

TIGECYCLINE

Daily schedule: 100 mg LD then 50 mg every 12 hours (2-hour infusion time)

+

FLUCONAZOLE

Daily Schedule: 600 mg LD then 400 mg every 24 hours (2-hour infusion time)

## Appendix 10. Antimicrobial therapy for nosocomial IAI in critically ill patients. (WSES recommendations)

Hospital-acquired extra-biliary IAIs

Critically ill patients (≥SEVERE SEPSIS)

Risk factors for MDR pathogens

PIPERACILLIN

Daily schedule: 8 g by LD then 16 g via continuous infusion or 4 g every 6 hours (4-hour infusion time)

+

TIGECYCLINE

Daily schedule: 100 mg LD then 50 mg every 12 hours (2-hour infusion time)

+

ECHINOCANDIN

caspofungin (70 mg LD, then 50 mg daily),

anidulafungin (200 mg LD, then 100 mg daily),

micafungin (100 mg daily)

OR

MEROPENEM

Daily Schedule: 500 mg every 6 hours (6-hour infusion time)

IMIPENEM

Daily Schedule: 500 mg every 4 hours (3-hour infusion time)

DORIPENEM

Daily Schedule: 500 mg every 8 hours (4-hour infusion time)

+

TEICOPLANIN

Daily Schedule: LD 12 mg/kg/12 h for 3 doses then 6 mg/kg every 12 hours (with TDM corrections/adjustments – PD target 20–30 mg/L)

+

ECHINOCANDIN

caspofungin (70 mg LD, then 50 mg daily),

anidulafungin (200 mg LD, then 100 mg daily),

micafungin (100 mg daily)

## Competing interests

The authors declare that they have no competing interests.

## Authors’ contributions

MS wrote the manuscript. All authors read and approved the final manuscript.
